# Indoors or Outdoors? An International Exploration of Owner Demographics and Decision Making Associated with Lifestyle of Pet Cats

**DOI:** 10.3390/ani11020253

**Published:** 2021-01-20

**Authors:** Rachel Foreman-Worsley, Lauren R. Finka, Samantha J. Ward, Mark J. Farnworth

**Affiliations:** 1 Brackenhurst Campus, School of Animal, Rural and Environmental Sciences, Nottingham Trent University, Nottinghamshire NG25 0QF, UK; lauren.finka@ntu.ac.uk (L.R.F.); Samantha.ward@ntu.ac.uk (S.J.W.); v1mfarnw@exseed.ed.ac.uk (M.J.F.); 2 Jeanne Marchig International Centre for Animal Welfare Education, Easter Bush Campus, Royal (Dick) School of Veterinary Studies, The University of Edinburgh, Midlothian EH25 9RG, UK

**Keywords:** cats, felines, indoor-only, indoor-outdoor, companion animal, *Felis catus*, management

## Abstract

**Simple Summary:**

Owners may consider many factors when deciding whether to provide an indoor-only or indoor-outdoor lifestyle for their cats. These include safety, mental and physical health, exposure to parasites or disease, and depredation of wildlife. This international study used a series of online surveys to explore the factors cat owners consider when deciding what lifestyle to provide for their cat, alongside investigating if owner and cat features are associated with greater odds of cats having indoor-only or indoor-outdoor lifestyles. Ten variables were found to be significant predictors of lifestyle. Owner features predicting a greater likelihood of cats being kept as indoor-only were being 26–35 years old, having multiple cats, living in city centres or urban areas, and living in the United States, Canada, Australia, or New Zealand. For cats, these features included being junior, having health issues, being pedigree, or having unknown pedigree status. Owner features predicting a greater likelihood of cats being indoor-outdoor were owners being 46–55+ years old or 56+ years old and having children (17 years old or under) living at home. For cats, features included being male and being mature or senior. Road traffic accidents were the major concern for owners of indoor-only cats in all regions surveyed. Owners who provided outdoor access predominantly indicated they did so for the mental wellbeing of their cat. These findings are important in understanding the considerations owners give to their cat’s lifestyle and identifying management trends and cat populations potentially at risk of compromised welfare due to unsuitable lifestyles.

**Abstract:**

Outdoor access for owned domestic cats (*Felis catus*) is a divisive issue. Cat safety, mental and physical wellbeing, infectious diseases, and wildlife depredation are cited as factors influencing owners; however, the degree of consideration each factor receives has not been quantified. This study (i) analysed which demographic variables are associated with greater odds of cats having indoor or outdoor lifestyles, (ii) identified which factors owners consider when making a choice on lifestyle and any regional variations, and (iii) identified if owners consider the different lifestyle options available and recognise their associated benefits. A series of online surveys were used for data collection. Binary logistic regression models were used to generate odds ratios assessing if demographic variables were significantly associated with cat lifestyle. Quantitative analysis of factors considered when deciding on cat lifestyle was accompanied by a thematic analysis of rich-text open-ended responses, providing nuanced insight into the rationale and elucidating additional factors considered. Of the demographic variables tested, 10/12 were significantly associated with lifestyle. Variables with higher odds of indoor-only lifestyles were owners being 26–35 years old, multi-cat households, junior cats, pedigree cats or unknown pedigree status, cats with health issues, living in city centres or urban areas, or living in the United States, Canada, Australia, or New Zealand. Variables with higher odds of indoor-outdoor lifestyles were owners being 46–55 years old or 56+ years old, households with residents 17 years old or under, male cats, and cats being mature or senior. Road traffic concerns were the most cited reason for keeping indoor-only cats across all global regions. The second-most cited reason varied regionally. For Europe, it was protection from people. For the USA and Canada, the reason was protection from wildlife, and for Australia and New Zealand, to prevent hunting. Indoor-outdoor cat owners cited most frequently the benefits to their cat’s mental health. Over two-thirds of owners did not consider the alternative lifestyle for their cat. These data give insight into the priorities of cat owners with regards to feline wellbeing, feline safety, and wildlife depredation, helpful for individuals or organisations working with human behaviour change. They provide evidence that the numbers of indoor-only cats are likely to rise with increasing urbanisation. Finally, the data identify cat populations who may be at risk of compromised welfare due to unsuitable, or under-researched, lifestyles.

## 1. Introduction

The provision of outdoor access for domestic cats (*Felis catus*) by their owners is a divisive issue [1,2] and likely influenced by cultural norms. In the United States of America (USA), 63% of domestic cats are kept entirely indoors [3]. In contrast, many European countries, including the United Kingdom (UK) [4] and Denmark [5], as well as Australia [6,7], typically provide owned domestic cats with outdoor access, in addition to allowing them to occupy the house. There is, however, a growing trend towards keeping cats exclusively indoors. The UK is seeing a rapid increase in the number of indoor-only cats, with the PDSA (People’s Dispensary for Sick Animals) producing estimates of 15% in 2011, increasing to 24% in 2015 [4], and a more recent UK study indicating 26.1% in 2019 [8].

At present, very little information exists regarding the factors that owners consider when deciding on a lifestyle for their cat, the weight owners assign to these factors, or if specific cat and owner demographic variables are associated with different lifestyles. This information could be of benefit to organisations, charities, or individuals to maximise the efficacy of human behaviour change incentives. It may also help to explain changes in cat management trends globally and predict how management trends may continue to change in the future.

For this study, a survey was distributed to an international population of current cat owners, exploring the rationales behind lifestyle choices for cats. To help inform survey questions, an initial overview of the current literature surrounding the factors that owners may consider when making a lifestyle decision for their cat was generated. This review is presented below.

### 1.1. Lifestyle Considerations

Hunting: Domestication of the cat was driven by their predatory nature, which was advantageous for pest control in early agricultural communities [9]. Since then, cats have experienced a relatively unique domestication process involving less intensive selection than animals such as dogs [10]. Consequently, most domestic cats have retained ancestral behavioural motivations, such as hunting drive irrespective of food provision [11]. Whilst hunting behaviour is still valued in some agricultural contexts, it is not typically valued by owners keeping cats as companions [12,13]. On the contrary, predatory behaviours are of growing concern as the numbers of domestic cats rise due to their impacts on native wildlife including birds, invertebrates, mammals, and amphibians [14,15]. The ecological impact of hunting on wildlife appears to vary between areas. More severe damage to ecosystems is thought to occur where cats represent an introduced predator and where wildlife has not evolved to avoid predation, such as Australia, New Zealand, or remote islands. In some such instances, cats have been credited as contributors to the extirpation or near-extirpation of species [16,17,18]. Consequently, some owners, at the behest of wildlife charities and veterinarians, opt to keep cats indoors to prevent hunting [19]. It is possible that concerns over impact severity may influence the consideration given to hunting by owners. For example, UK cat owners generally disagree that cats are harmful to wildlife, regardless of the predatory behaviour of their cat [20]. In Australia and New Zealand, however, 62% and 51% of cat owners, respectively, agree that predation is problematic [13].

Cat safety: Outdoors, road-traffic accidents (RTAs) are likely a major concern to cat owners. A UK study found the major cause of mortality for cats brought into a veterinary clinic was trauma, 60% of which were identified as RTAs [21]. An estimated 12% of cats in Cambridgeshire, UK had been involved in an RTA and survived [22], suggesting a higher percentage of cats are involved in RTAs in total when also accounting for mortalities. Additional outdoor risks include attacks by humans, and where feral cats are considered as pests and lethally controlled, domestic cats may risk being indiscriminately killed through poisoning [23] or other pest control methods. There is also the potential to consume toxins such as pesticides, insecticides, anti-freeze, or toxic outdoor plants. Indoors, cats may ingest toxic substances, such as cleaning products, houseplants or flowers, medicines, or toxic food substances [24], or risk electrocution from household appliances. Both indoors and outdoors, there is a possibility of injuries or bites from wild and domesticated animals, including other cats [25].

Physical health: A positive correlation between obesity in cats and an indoor-only lifestyle has been demonstrated, with potential mechanisms cited as being a reduced physical activity, greater consumption of food through boredom, and lack of enrichment [26,27,28]. Obesity, reduced activity, and toileting exclusively indoors have also been associated with increased risk of feline urological syndrome (FUS) [29]. It is possible some owners may utilise outdoor access as a weight management tool. Outdoors, however, cats are at greater risk of contracting diseases such as feline immunodeficiency virus (FIV), feline leukaemia virus (FeLV), ringworm and cat flu, or parasites due to their contact with wildlife and other domestic cats [30]. Owners may wish to reduce these risks to improve welfare or prevent associated veterinary treatment or zoonotic transmission. Additionally, owners of cats with contagious diseases may choose to house their cats indoor-only to prevent disease transmission to other cats.

Mental well-being: Many behavioural needs of cats, such as hunting, territorial patrolling and marking, roaming, and climbing may be more readily met in an outdoor environment [11]. Whilst owners may instead aim to meet their cat’s behavioural needs indoors, studies suggest many cat owners may not provide adequate levels of enrichment to ensure high welfare for their cat [31,32,33]. Insufficient levels of enrichment and the inability to avoid stressful human–social environments indoors [34,35] may contribute towards the comparatively higher levels of undesirable and sickness behaviours observed in indoor-only cats, compared to indoor-outdoor cats [5,8,36,37,38], although it has been reported in one instance that indoor-outdoor cats may display more undesirable behaviours [31]. With regards to owner attitudes, an Australian study revealed most indoor-outdoor cat owners felt wandering was natural and necessary for cats to be ‘happy’ [19]. In the USA, owners were mixed in their response when asked if cats needed time outdoors to be ‘happy’ [31]. In a Brazilian study, just 7.5% of owners felt it was necessary for cats to have outdoor access [39]. Whilst owners may perceive some aspects of outdoor access as beneficial to cat mental health, it must be considered that owners interpret other aspects as detrimental, lest their cats perceive potential dangers, novel environments, sights, and sounds or territorial conflicts with conspecifics negatively.

### 1.2. Aims and Objectives

- Identify if different owner features or cat demographics are associated with greater odds of cats having an indoor-only or indoor-outdoor lifestyle;
- Elucidate the extent to which factors identified from the literature influence owners when making lifestyle decisions for their cat, and what proportion of owners consider the different lifestyle options available;
- Establish major narrative themes around owner decision making.

## 2. Materials and Methods

### 2.1. Survey Creation and Distribution

An initial online survey (part 1) for cat owners was distributed in English via social media, predominantly Facebook and Twitter, between February 2019 and April 2019. It purposively sampled cat owners using relevant social media groups and cat-related hashtags. To participate, respondents needed to be 18 years old or over and the current owner of at least one cat which did not live exclusively outdoors. The survey comprised of the following sections: owner demographics, cat demographics, cat health and behaviour (including both sickness and undesirable behaviours), cat personality, cat lifestyle (indoor-outdoor or indoor-only) and the basic rationale for said lifestyle, home environment inclusive of basic provisions and enrichment and social behaviour with adults, children, and cats and dogs within the household. Questions consisted of multiple and single choice questions, Likert scales, and open-ended text-based questions. The survey was developed as part of a wider study looking at cat management and welfare, of which a subset of data is considered for the purposes of this paper.

Participants who responded to the initial survey (part 1) and had provided an email address were sent a second survey (part 2) exploring the rationale for choosing an indoor-only or indoor-outdoor lifestyle for their cat. Through a series of questions, owners were asked to identify factors influencing their decision and the strength of their consideration using Likert scales. Part 2 also established if owners of indoor-only cats had considered outdoor access and vice versa and identified the strength of consideration given to aspects of the alternative lifestyle. Respondents were encouraged to leave as many details as possible in open-ended questions. Owners of both indoor-only and indoor-outdoor cats answered the same questions, reworded to be appropriate to each group. Responses from part 1 and part 2 were matched using email addresses, so demographic data could be associated with rationales.

Ethical approval was given by Nottingham Trent University School of Animal, Rural and Environmental Sciences Research and Ethics Committee on the 11th December 2018 (ARE843).

### 2.2. Data Cleaning

Owners who indicated they intended to change their cat’s lifestyle were excluded from the analysis (*n* = 34). These owners detailed reasons such as recently acquiring their cat, recently moving to a new house, or having a cat they deemed as currently too young to roam, including those awaiting neutering. Owners providing different answers for their cats’ lifestyles for part 1 and part 2 of the survey but who had not indicated they had changed their cat’s lifestyle were excluded to avoid reporting errors by respondents (*n* = 16). Finally, those who had categorised their cat as having one lifestyle but provided contradictory comments were excluded (*n* = 43), e.g., one owner indicated their cat was indoor-only but commented, ‘The cat does have some supervised time outside […]’.

Owners were categorised into three major regions—Europe, USA and Canada, and Australia and New Zealand. Other regions were excluded from analyses due to low sample sizes (*n* = 154). These regions were chosen to compare attitudes towards cat management which might be influenced by variations in local legislation and recommendations from regional feline welfare charities [40,41,42].

### 2.3. Data Analysis

Responses were divided into populations of owners providing their cats with either an indoor-only or indoor-outdoor lifestyle. A combination of Microsoft Excel (Version 2002, Microsoft, Washington, DC, USA) and IBM SPSS (Version 26, New York, NY, USA) was used to generate descriptive statistics exploring the demographics of cats with different lifestyles. Descriptive statistics were also used to gain insight into the frequency of responses from quantitative multiple-choice questions.

Open-ended responses were read in their entirety by the lead author, RFW. A portion of open responses directly reflected the multiple-choice answers provided and was coded as such. Responses that did not fit existing answers were classed as ‘other’ reasons within quantitative analyses. These ‘other’ responses were taken forward to an additional qualitative thematic analysis and coded as new semantic themes, using the six-phase methodology defined by Braun and Clarke [43]. In keeping with qualitative methods and considering that responses were optional and therefore not balanced amongst key owner and cat demographic variables, themes were not quantified. Themes and example responses are instead provided to allow insight into the wide range of factors owners may consider when choosing their cats’ lifestyle, alongside the depth of thought and emotion behind these considerations.

### 2.4. Odds Ratios

Demographic variables hypothesised to have biological relevance to owner decisions on cat lifestyle choice were explored using binary logistic regression modelling. Odds ratios were calculated to elucidate if specific variables predicted a greater likelihood of an indoor-outdoor or indoor-only lifestyle. Three models were produced, each with ‘lifestyle’ as the response variable.

Model one explored associations between lifestyle and owner social features, with explanatory variables of owner gender, owner age, and the number of other cats, dogs, and children (17 years old and under) in the home. It was hypothesised that owners of different generations with differing levels of social intensity within their homes may make different lifestyle choices for their cats.

Model two’s explanatory variables were cat features of age, sex, ongoing health issues, and pedigree status. It was hypothesised owners may make decisions based on the specific characteristics of their cat and what they deemed to be the most appropriate lifestyle for that individual. Cat ages were categorised into life stages for analysis, based on definitions provided by Vogt et al. [44], which are as follows: kitten, 0–6 months old; junior, 7 months–2 years old; adult, 3–6 years old; mature, 7–10 years old; senior, 11–14 years old; super senior, 15+ years old. Due to the small numbers of super senior cats in the sample, these cats were grouped with senior cats to create a senior category of 11+ years old. Neutering, microchipping, vaccinating, and declawing were not deemed to be biologically relevant explanatory variables for this model. It was deemed more plausible that lifestyle choice would impact the decision of owners to provide such treatments to their cats, rather than vice versa.

Model three explored geographic features and consisted of explanatory variables of the global region, area type, e.g., rural, urban, etc.; and dwelling type, e.g., flat/apartment, detached house, etc. It was hypothesised that differing cultural norms may impact lifestyle choices between regions, and that area and dwelling type may influence owners based on the availability and quality of outdoor access.

Reference categories were set as the normative categories. For owner gender, cat sex, owner and cat age, region, area, and dwelling type these were the variable category with the largest portion of respondents. For the presence of other cats, dogs, under 17-year-olds, health issues, or pedigree status, the reference categories were set as ‘no’.

Due to small group sizes making for unbalanced categories, excluded from the analysis were owners who had indicated ‘prefer not to say’ for either age or gender, owners identifying as ‘other’ for gender, owners unsure of their cats’ sex or age, owners living in movable homes such as motorhomes or barges, one owner who indicated they lived in a Souterrain (cellar), and kittens <6 months old. If responses were excluded, they were excluded across all three models. In total, 4909 samples out of the original 5129 were analysed.

## 3. Results

From the first part of the survey, 5129 responses were included. Part 2, exploring lifestyle rationales in more depth, was emailed to a subsample of those participants (2581/5129) and returned by 459/1071 of indoor-only respondents (response rate 46.4%) and 595/1510 of indoor-outdoor respondents (response rate 39.4%). As not every question was answered by all participants due to survey routing, the number of respondents is detailed with each result presented within this section.

### 3.1. Demographic Results

Of the initial 5129 survey respondents (prior to those excluded for the odd ratios analysis), most respondents were female (89.1%), 26–35 years old (28.2%), had no children under 17 years old living with them (80.4%), owned more than one cat (55.3%), and had no dogs (81.8%). Most respondents lived in Europe (76.2%), falling across 36 European countries in total, although the majority were UK-based (80.3%). A full breakdown of owner demographics can be seen in Table 1, divided into populations of owners that provided either indoor-only or indoor-outdoor environments for their cats.

The 5129 cats answered for were relatively evenly split between sex, with 50.6% being female. The majority were neutered (96.8%), microchipped (79.0%), up to date with relevant vaccinations by the owner’s definitions (75.4%), not declawed (97.9%), and had no health problems (83.4%). A full breakdown of cat demographics and the split between indoor-only and indoor-outdoor cats can be seen in Table 2.

### 3.2. Variables as Predictors of Lifestyle (Odds Ratios)

Of the 12 major variables tested across the three described models, 10 were found to be significantly associated with cat lifestyle. Full details can be found in Table 3, Table 4 and Table 5, whilst a summary is provided below.

Variables with greater odds of cats having an indoor-only lifestyle were owners who were 26–35 years old (*p* = 0.001, odds ratio (OR) 0.765) when compared to those 36–45 years old, cats in multi-cat households (*p* < 0.001, OR 0.768) compared to single cat households, junior cats (*p* < 0.001, OR 0.656) when compared to adult cats, pedigree cats (*p* < 0.001, OR 0.441) or those whom owners were unsure of their pedigree status (*p* = 0.004, OR 0.707) compared to non-pedigree cats, cats with health issues (*p* < 0.001, OR 0.596) compared to cats with no health issues, living in city centres (*p* < 0.001, OR 0.442) or urban areas (*p* = 0.001, OR 0.730) when compared to suburban areas, and living in the USA and Canada (*p* < 0.001, OR 0.093) or Australia and NZ (*p* = 0.001, OR 0.510) when compared to living in Europe.

Variables found to have greater odds of being associated with an indoor-outdoor lifestyle were owners being 46–55 years old (*p* = 0.006, OR 1.281) or 56+ years old (*p* < 0.001, OR 1.499) when compared to those 36–45 years old, owners with children (17 years old or under), living at home (*p* < 0.001, OR 1.707) when compared to those without, cats being male (*p* = 0.016, OR 1.155) compared to being female, and cats being mature (*p* = 0.047, OR 1.179) or senior (*p* < 0.001, OR 1.445) when compared to adult cats.

### 3.3. Lifestyle Choice Rationale

#### 3.3.1. Indoor-Only Owners

Of owners of indoor-only cats, 73.1% (1538/2104) indicated the lifestyle was their preference, 18.7% (393/2104) indicated they did not have the option to provide their cat with outdoor access, and 8.2% (173/2104) reported their cat chose not to go out even when given the choice. As seen in Table 6, a total of 85% (1133/1333) of the major reasons given for choosing an indoor-only lifestyle pertained to cat safety, not inclusive of additional reasons provided for the ‘other’ category.

Protection from traffic was the largest influencing factor for owners across all three regions. It was cited as the major reason for choosing an indoor-only lifestyle by most indoor-only owners at 58.7% (782/1333), and 98.7% (1435/1454) of indoor-only owners were influenced by traffic to some extent when making their decision, with 86.7% (1261/1454) saying traffic strong factor in their decision. The second major reason for indoor-only owners choosing this lifestyle varied between regions. Owners in Europe cited it to be protection from people (18.1%, 117/645), the USA and Canada cited protection from wildlife (19.9%, 126/634), and owners in Australia and New Zealand cited it was to prevent cats hunting (29.6%, 16/54).

Of indoor-only owners, 71.5% (328/459) said they had not considered the alternative of an indoor-outdoor lifestyle. Of indoor-only owners who did consider the alternative lifestyle, 35.3% (46/131) cited the major reason they would change would be the potential benefits to mental health. Overall, 96.1% (126/131) of indoor-only owners considering an indoor-outdoor lifestyle considered potential mental health benefits of outdoor access in some capacity, with 57.3% (75/131) of owners considering this strongly. More details can be found in Table 7.

#### 3.3.2. Indoor-Outdoor Owners

For indoor-outdoor owners, the benefit of outdoor access to mental health was the major cited reason for allowing cats outdoor access at 38% (226/595). The second most cited reason was that the cat indicates they want to go outside at 32.9% (196/595). A global breakdown for indoor-outdoor owners is not provided as it is for indoor-only owners, as although part 2 of the survey was distributed to owners in all regions, all respondents resided in Europe.

Of indoor-outdoor owners, 70.8% (421/595) said they had not considered the alternative of an indoor-only lifestyle. Of those who did, traffic was again considered a risk. Of indoor-outdoor cat owners who contemplated an indoor-only lifestyle, 96.6% (168/174) considered traffic, with 74.1% (129/174) stating this was a strong consideration. Protection from traffic was the most cited reason owners would switch to an indoor-only lifestyle at 45.8% (80/174). More details on owners who considered an indoor-only lifestyle can be found in Table 8.

### 3.4. Thematic Analysis of Responses

#### 3.4.1. Rationales of Indoor-Only Cat Owners

In addition to the reasons provided within the survey, as detailed in Table 6 and Table 7, six additional themes were identified from open-text responses. These are as follows: protection from traffic; protection from people; protection from other animals (including wildlife* and other cats); cat has health issues; to protect wildlife; protection from illness*; to prevent getting lost*; acquisition requirement/recommendation*; personality unsuitable*; pedigree cat*; cat has no previous outdoor experience*. Themes without an asterisk were included within the initial survey, whilst those marked with an asterisk (*) were identified from open-text responses. Table 9 highlights example quotes from owners used to create these themes.

Theme 1, Protection from traffic: Protection from traffic was the most common consideration influencing owners to keep cats indoors. Primarily, owners focussed on the risk of injury or death. Some owners indicated this fear was due to prior experience. Traffic concerns appeared so strong that an absence of traffic may be enough for some owners to change to an indoor-outdoor lifestyle.

Theme 2, Protection from people: Owners were concerned that people may cause intentional harm to their cat. Comments referenced local incidents or specific neighbours who had displayed such behaviours previously. Theft was an additional concern for pedigree and non-pedigree owners, but for different reasons. Owners of pedigree animals were concerned their animal would be targeted due to their unique appearance and resale or breeding value. Owners of non-pedigree cats mentioned concerns over their cat being taken as bait for dogfighting.

Theme 3, Protection from other animals: Concerns regarding interactions with other animals could be divided into those pertaining to cats (both owned and feral), local wildlife *, and dogs *. Encounters with other cats were viewed as dangerous due to fighting or disease transmission. Fighting was deemed to have detrimental physical and mental implications. It was of specific concern for those with timid cats who wanted to avoid their cat being ‘bullied’, or of owners with older cats who feared their animal would be unable to defend themselves. Owners with local feral colonies nearby were additionally concerned about these cats being higher risk disease vectors. More on the concerns of disease transmission is discussed in theme 6. With regards to wildlife, owners feared their cat may become a victim of predation and listed large mammals or birds as potential predators. Snakes were also mentioned specifically, alongside their potential to injure or kill cats and previous bad experiences. Comments pertaining to the potential dangers of dogs predominantly focussed on owned dogs that may attack cats. In some instances, these dogs were known to the owner and were deemed a particular risk.

Theme 4, Cat has health issues: Owners felt specific medical issues made it more dangerous for their cat to be outside. FIV was often mentioned explicitly. Some owners gave no further explanation other than to say their cat was FIV+, whilst others detailed their concern for the health of their animal, disease transmission to other cats, or both. Owners were also concerned outdoor access would mean being unable to control medical issues due to being unable to monitor what the cat was ingesting or being unable to give medication when required.

Theme 5, To protect wildlife: Owners viewed an indoor-only lifestyle as an easy way to prevent hunting. This was typically to prevent damage to local bird populations, although some comments additionally mentioned reptiles or small mammals.

Theme 6, Protection from illness (*): Several illnesses were mentioned as potential threats, with many of them such as flu, FIV, or FeLV considered infectious. Owners of cats with ongoing medical conditions had specific concerns about their cats contracting further illness (as discussed in theme 4). Owners highlighted concerns over parasites such as fleas, ticks, or worms, however, in many instances, the focus was not on the welfare of the cat, but rather the owner’s discomfort. Owners felt parasites were dirty or unpleasant and something that should not be brought into the home. Owners also acknowledged the inconvenience and expense of the requirement to upkeep preventative treatment of parasites for cats with outdoor access. Additionally, owners highlighted concerns about cats consuming dangerous plants they would not encounter indoors or encountering poisonous substances (e.g., anti-freeze or pesticides) neighbours may use and leave in their gardens.

Theme 7, Prevent getting lost (*): Owners indicated that their cats were kept indoors to prevent them from getting lost. It was not typically cited if this concern was for their cat’s welfare or their own, or if they had attempted to allow their cat outdoors. Some owners suggested they had let their cat out, and the cat returned to a previous home in which they lived. A few owners alternatively used the phrase ‘run-away’, suggesting they feel their cat may intentionally not return if given the opportunity.

Theme 8, Acquisition requirement/recommendation (*): The opinions of other people were often taken into consideration, particularly those from the place owners had acquired their cat. Adoption centres were frequently cited as influencing owner choice of lifestyle, with some rescue organisations recommending indoor-only lifestyles for specific cats in their care based on their history and temperament. Other rescue organisations appeared to have a blanket policy on all cats being kept indoors. Breeders of pedigree animals also frequently required cats to be indoor-only. Whilst some owners alluded to these being recommendations, in some instances, owners reported both breeders and rescue shelters requiring them to sign a contract committing to keeping their cat indoors.

Theme 9, Unsuitable personality (*): Some owners felt their cat’s temperament made them unsuitable to go outdoors. Some felt their cat’s temperament may put them at a greater risk of harm outdoors, such as skittish cats or over-friendly cats. Other owners seemed to feel that the experience of being outdoors would be detrimental to the cat’s mental welfare, especially owners of cats who intensely disliked other cats or cats deemed to be timid/shy/anxious. Some owners seemed to indicate they had attempted some form of outdoor access off which they had based their decision, whilst other owners made the decision without trying any form of outdoor access beforehand.

Theme 10, Pedigree cat (*): In addition to the concerns over theft, as presented in theme 2, pedigree cats were often kept indoors as their temperaments were deemed unsuitable to have outdoor access. Numerous breeds were cited as being incapable of looking after themselves outdoors. Other owners believed their cat had no desire or need to go outdoors and has been bred to be indoor-only. A small number of owners felt their breed was unsuitable to go outdoors due to physical attributes, e.g., hairless breeds being unable to keep warm. Less often, owners were using their cats for breeding and aimed to prevent unwanted pregnancy.

Theme 11, No previous outdoor experience (*): Owners of cats who had previously been kept as indoor-only did not want to change that lifestyle to indoor-outdoor. These cats were typically not obtained by their owners when they were kittens. When acquired as adults, owners felt their cats lacked the experience needed to stay safe whilst roaming and so were better off staying indoors.

#### 3.4.2. Rationales of Indoor-Outdoor Cat Owners

For indoor-outdoor cat owners, in addition to the five themes provided within the survey questions, five further themes were identified from open-text responses. These were ‘beneficial to mental health’, ‘beneficial to physical health’, ‘cat indicates they want to go outside’, ‘cat toilets outside’, ‘pest control’, ‘enrichment*’, ‘previous outdoor access*’, ‘social opportunity*’, ‘safe outdoor space*’, ‘multi-cat household*’, and ‘natural*’. Table 10 highlights example quotes from owners used to create these themes.

Theme 12, Beneficial to mental health: Alongside the thinking that outdoor access was beneficial to mental health, it was felt that confining a cat to the indoors could have negative impacts. Some owners detailed having experienced this with their current or previous cat. The impact of being confined indoors on the cat’s mental health was often described as causing stress, depressive states, or states of (sometimes extreme) agitation. Many owners felt that the outdoors did not just prevent negative mood states, but also promoted positive experiences. This is discussed further in theme 17.

Theme 13, Beneficial to physical health: Owners who felt the outdoors was beneficial to physical health recognised that the opportunity for exercise was good for weight management. Owners also mentioned the overlap between physical and mental health. Poor mental health and stress were cited as causing general sickness behaviours, such as vomiting or poor coat condition. Other owners detailed how the stress caused or exacerbated existing conditions, such as cystitis.

Theme 14, Cat’s choice: Many owners simply let their cat decide whether they wanted outdoor access. Autonomy and choice were recognised as mentally beneficial for cats in addition to the outdoor access itself. This was so important to some people that they allowed outdoor access even if they would have preferred otherwise. Not giving cats a choice was often deemed as cruel or unfair. Additionally, some owners seemed to appreciate the fact that their cat lived with them through choice because they had the opportunity to leave yet did not take it.

Theme 15, Cat toilets outside: Mentions of toileting habits were predominantly from the perspective of the cats who preferred to do so outside rather than using the litter tray, or in some instances, would only toilet outside. Some owners did, however, mention they preferred their cat to toilet outside.

Theme 16, Pest control through hunting: Whilst many owners kept cats indoors to prevent hunting, as discussed in theme 5, some owners found this trait to have positive utility in terms of pest control. Hunting as a form of enrichment was also identified as beneficial and is discussed more in theme 17.

Theme 17, Enrichment (*): Owners often felt the outdoors provided good enrichment for their cat to keep them entertained and stimulated. Some people detailed that they had purposefully added objects into the garden to accentuate this further. Often, it was felt that this outdoor enrichment was unique and could not be readily replicated indoors, specifically with regards to weather. Sunshine was viewed as a positive experience for many cats who appeared to actively enjoy spending time in it. Fresh air was mentioned as being enjoyable from a cat’s perspective, but owners also indicated they felt it beneficial. Cats were detailed as avoiding less favourable weather, such as rain or cold temperatures, but this was usually a choice that the cat was free to make.

Sub-theme of Theme 17: Opportunity to hunt—The opportunity to hunt was often viewed as a natural and instinctive behaviour which could readily be provided for outdoors should the cat wish. Owners did not necessarily encourage this behaviour but accepted it as beneficial to the cat’s wellbeing for them to have the opportunity. Some owners had aversions to hunting but appeared to feel their cats’ wellbeing outweighed this.

Theme 18, Previous outdoor access (*): Many owners obtained adult cats with previous outdoor experience and so felt they did not want to deprive them of the outdoor access they had been used to. Cats who were strays, feral, or farm cats were specifically mentioned as these cats were used to spending large portions of their time outdoors. Some owners alluded to keeping these cats indoors temporarily to detrimental effect.

Theme 19, Social opportunity (*): The opportunity for social interaction with people and conspecifics outside of the immediate household was recognised as beneficial. Cats were detailed as enjoying interacting with neighbours, and owners appreciated how this brings happiness to the neighbours in turn. It was also felt to be unfair to not allow cats to have the opportunity to socialise when the owners were not at home. Cats were also reported to spend time interacting positively with other cats in the neighbourhood.

Theme 20, Safe outdoor space (*): The dangers cited by the owners of indoor-only cats, such as traffic or wildlife, were also acknowledged by the owners of indoor-outdoor cats, yet many owners felt the area they lived in was safe enough to mitigate the risks of injury or death sufficiently. For those who felt their area was safe enough to allow their cat outside, it was unclear what they would do should they have to move, with some owners acknowledging they might reconsider providing outdoor access in such circumstances. However, some owners felt the outdoors was of such benefit to their cat that they ensured their property was safe enough to allow outdoor access when they were looking for a home.

Theme 21, Multi-cat household (*): The management of cats within a multi-cat household was deemed to be easier by allowing outdoor access. Many cats lived in multi-cat households where the lifestyle of previously obtained cats determined the focal cat’s subsequent lifestyle. In instances where the focal cat had joined a household which already contained cats with outdoor access, owners felt that unequal treatment was unfair and that the cats themselves may feel so too. Additionally, the extra space provided outdoors was cited to be beneficial for allowing cohabiting cats to have time away from one another. It was felt that outdoor space reduced the amount of physical conflict and aggression between cats of the same household.

Theme 22, Naturalness (*): The term ‘natural’ was frequently used in explaining why outdoor access had been chosen. This was seen to encompass many of the previously discussed themes around mental and physical benefits, enrichment such as climbing and exploring, as well as the need to hunt. The unique domestication of cats and fluidity of individual cats’ socialisation was also mentioned to highlight the ‘nature’ of cats and as a reason to allow outdoor access.

## 4. Discussion

Overall, 41% of cats within this study were indoor-only. Differences were seen among the three global regions—at 30.2% in Europe, 80.6% in the USA and Canada, and 42.2% in AUS and NZ. Region was found to have a significant impact on lifestyle, with cats comparatively much less likely to be indoor-outdoor in the USA and Canada (OR 0.093) and AUS and NZ (OR 0.510) than in Europe. The proportions of cats kept indoors in Europe in this study were not too dissimilar to others. In the UK, 26.1% of cats were indoor-only [8]; in Denmark, 16.8% of cats were indoor-only; [5] and in France, 34% were indoor-only [45]. For the USA and Canada, results in this study showed a higher percentage of indoor-only cats compared to a reported 63% [46] and 60% [31] for the USA, or 56% in Canada [47]. For AUS and NZ, it has been reported that 44% [48] or 46.5% [7] of cats in Australia are indoor-only, whilst in Melbourne specifically, it was reported to be 23% [49]. In New Zealand, it has been reported that 10.7% of cats were indoor-only at all times [50], whilst 26% were indoors during the night [51]. Although our study broadly concurs with others there are notable differences in proportions of indoor cats within specific regions. It is likely the intra-region variation amongst studies arises due to the grouping of regions in this study, where previous studies typically focus on a single country or state within a country.

### 4.1. Safety

Safety, in some regards, was the primary motivating factor for keeping cats indoors across all three regions (USA and Canada, 84.3%; Europe, 88.2%; and Australia and New Zealand, 53.7%). The motivation for owners wanting to keep their cat safe seemed to be both a concern for the welfare of their cat and protecting themselves from the emotional harm of losing their cat to fatal incidents. Safety concerns have been acknowledged in other studies. A UK study found 63% of UK owners with indoor cats felt it was unsafe for their cat to be outdoors [52]. An Australian study found 75.4% of cat owners felt keeping cats contained was important to protect them from injury [33]. Whilst a New Zealand study found 45% of people who kept their cats indoors at night did so due to safety [53]. These differences in numbers may arise due to variations in the owner populations being studied. Both the Australian and New Zealand studies included owners who allow their cats to roam in some capacity and such owners are perhaps less likely to be concerned over safety than the owners of indoor-only cats.

### 4.2. Road-Traffic Accidents

The greatest influencing factor for an indoor-only lifestyle, which was consistent across global regions, was protection from RTAs. Concerns over RTAs have been indicated elsewhere. Of UK indoor-only cat owners who deemed the outdoors to be unsafe, 83% felt this way due to traffic concerns [52]. However, incidences of RTAs, in the UK at least, appear relatively low. Whilst fatal RTAs are difficult to quantify because they are typically not reported, and record keeping by local authorities and veterinary practices vary, several UK studies have aimed to estimate these figures. One study found only 4.2% of cats registered to VetCompass and presented to the emergency, out-of-hours practices in the UK between January 2012 and February 2014 had been involved in an RTA [54]. In Cambridgeshire, UK, an estimated 12% of cats had survived an RTA [22]. With these veterinary studies, it must be considered that RTA numbers are likely to be higher because deceased animals are not likely to be presented to practices. A longitudinal cohort study of 1264 UK cats negated this bias of not reporting fatal incidents and found that, within the first year of life, 3.9% of cats were found to be involved in RTAs, with 71.4% of these being fatal [55]. These UK-based figures are unlikely to be applicable to other countries, or even regions within the UK different from the studied area, due to differing densities of free-roaming cats, varying levels of traffic, or traffic speed. Consequently, more research into the RTA rates in different regions is required to help owners better understand the risks of providing an indoor-outdoor lifestyle.

Despite these overall low incidence rates, many previous studies have identified RTAs to be a major, or leading, cause of accidental death for younger pet cats specifically [21,22,56,57]. The increased risk for younger cats is likely due to a combination of factors such as a lack of experience and higher energy levels, resulting in a greater propensity to roam [57]. It has also been found that older cats are less likely to engage in risk-taking behaviours, including crossing roads [58]. Results from this study suggest owners may recognise that potentially risky behaviours may be more common in junior cats because this age group had greater odds of being kept as indoor-only cats compared to adult cats. Given the energy levels of, and stimulation required for younger cats, it is therefore particularly important that sufficient enrichment is provided within the home. In contrast, senior cats were the most likely age group to be provided with outdoor access. In free-text responses, older cats were detailed as only utilising garden spaces rather than roaming freely. For example, ‘Eldest [cat] is 11 years [old] and goes out unsupervised twice a day but remains in the garden’ and ‘Now he [cat] is older he never leaves my garden’. For older cats with previous experience outdoors particularly, it is promising that the recognition of this lower-risk outdoor behaviour may alleviate owner safety concerns over RTAs and make them more amenable to providing outdoor access.

### 4.3. Urbanisation

It is known that the number of indoor-only cats is rising, and it has been theorised that this may, in part, be due to increasing urbanisation. This theory is supported by the findings of this study, which indicated city or urban-dwelling owners and those living in flats or apartments are significantly more likely to have an indoor-only cat. Alongside owners not having outdoor space available, increased traffic in these urbanised areas is likely to be a contributing factor to the number of indoor-only cats, given the high level of safety concerns reported. Despite RTA fears in urban areas, and some indoor-outdoor owners only allowing outdoor access for their cat because they felt they lived in an appropriately quiet area with an absence of traffic (theme 20), the concerns over increased RTAs within built-up areas may be unfounded. One study found no significant association between area (urban/rural) and higher RTA mortality [57]. Whilst a second did find differences in RTA prevalence between areas, it was cats living in rural areas that seemed to be at increased risk when compared to cats within urban environments [55]. More detailed insights as to how, where, or when RTAs occur, including the time of day, could mean that owners in lower-risk areas are able to make more on-balance decisions, comparing the risks of outdoor access and any individual needs of their cat.

### 4.4. Variation between Regions

The second most cited reason for keeping cats indoor-only varied throughout the three regions. In the USA and Canada, it was for protection from wildlife (19.9%). In Europe, it was protection from people (18.1%). In Australia and New Zealand, it was to prevent cats from hunting (29.6%). This difference between regions could be due to variation in geography, urbanisation, and local wildlife. Respondents from Europe were predominantly UK-based (80.3%). The UK is densely populated and highly urbanised when compared to many regions within the USA, Canada, Australia, and New Zealand, which all have large, sparsely populated areas. Therefore, it is reasonable that owners in Europe have urban-centric concerns, whilst in the USA, Canada, Australia, and New Zealand, concerns are typically nature-orientated. The differences in concern for wildlife in the USA, Canada, Australia, and New Zealand may be explained by the types of wildlife found between the regions, as well as whether predators are endemic or not.

In the USA and Canada, large predators are commonplace. The presence of larger predators such as coyotes, eagles, or bears may mean prey species have adapted better to avoid predation, dampening the effects of depredation by cats and meaning cats are at risk of predation themselves. In Australia and New Zealand, there are no large predators, although poisonous insects or snakes may still pose a risk to cats. The absence of predators makes local wildlife particularly susceptible to cat depredation, and this ecological niche has made it easy for cats to reproduce and survive. Consequently, in Australia and New Zealand, there are large feral populations which have been estimated as being more numerous than owned cat populations [59], and cats are classed as an invasive species [41]. Many studies in Australia and New Zealand have investigated the attitudes of both cat owners and non-owners towards wildlife depredation and have repeatedly found it is a concern for both [33,60,61]. The management of cats has previously been found to reflect this concern, with many owners in these regions restricting outdoor access entirely or at certain times of the day [33,51], echoing the findings of this study.

### 4.5. Pedigree

Pedigree cats were more likely to be kept indoors than non-pedigree cats. Pedigree animals were described as ‘stupid’, ‘dopey’, or lacking ‘common sense’, and owners stated they were ‘not designed for the outdoors’ or had been bred to be indoor-only. Despite these concerns, the authors of this paper find no evidence to suggest different breeds may be suited to an indoor-only or indoor-outdoor lifestyle. It is possible that specific conformations could limit the ability of some pedigree breeds to survive outdoors. For example, brachycephalic breeds, such as Persians or Exotics, may suffer from respiratory issues [61] and struggle to eat or chew due to shortened muzzle lengths and dental abnormalities [62], which could impair activity levels and hunting behaviours. Hairless breeds, such as Sphynx cats, may struggle to regulate their temperature in colder climates. Whilst these phenotypic variations may reduce life-expectancy in unowned cats, it is not known if they would substantially impact behaviour or welfare in cats provided with shelter, food, and veterinary care, who also have outdoor access. Indeed, it has been tentatively found that pedigree cats are less likely to be in RTAs than non-pedigree cats, although sample sizes within the study were small [22]. It was posited this could be due to more time-restricted outdoor access of pedigree cats, or that owners may spend more close contact time with pedigree animals than non-pedigree, which mayin turn impact their behaviour outdoors. As evidence is emerging that behaviour may vary between breeds, in terms of social behaviour, activity levels, and temperament [63,64,65,66,67,68], it is reasonable to assume that variation in temperament may impact suitability for different living conditions. Further research into how breed-specific differences influence welfare across different lifestyles is therefore warranted.

### 4.6. Mental Wellbeing

Both indoor-only and indoor-outdoor owners felt outdoor access was beneficial to mental wellbeing, seemingly as it readily allows for the expression of natural behaviours. Outdoor-specific enrichment included weather (theme 17), hunting opportunities (theme 17), toileting preferences (theme 15), and socialisation with other cats and people (theme 19). Some owners detailed how they provided additional enrichment for their cats in their outdoor spaces. At present, no research has been conducted on the quality of outdoor environments cats have access to and their implications for cat welfare. It is possible that welfare differences may arise between cats with outdoor access living in rural environments with few other domestic cats, large open areas, and abundant wildlife such as bird and rodents compared to cats living in urban environments with dense cat populations and those with either basic or enriched outdoor spaces provided by their owners.

### 4.7. Physical Health

Potential benefits to the physical health of outdoor access were strongly recognised by both indoor-outdoor and indoor-only owners. Comments alluded to the opportunity for exercise, and how obesity might be mitigated through outdoor access if it increases overall activity levels in indoor-outdoor cats compared to their indoor-only counterparts. These owner views are consistent with literature that suggests an indoor-only lifestyle is a risk factor for feline obesity [27,68]. Obesity was recently cited by UK vets as the major health concern for owned pet cats [69], with obese animals being more likely to suffer from additional ailments such as arthritis or diabetes [70]. More research into the activity levels of indoor-only versus indoor-outdoor cats, or the exercise opportunities of indoor-only cats, could help with management strategies to ensure healthy weights and could be a cheap, easy, and non-invasive way for owners to improve their cat’s welfare.

A growing body of literature also suggests that stress-related illnesses, such as lower urinary tract signs, are typically more prevalent in indoor-only cats [71,72]. Although anecdotal, some owners did appear to notice improvements in their cat’s physical health when cats were given outdoor access after a previous restriction, and this was sometimes linked to improvements in mental health (theme 13). It might therefore be that affording outdoor access to cats with some pre-existing conditions could help to alleviate them. Despite this, pre-existing health conditions were found to be a significant predictor for cats being kept indoor-only. Whilst the frequency of specific pre-existing health conditions was not quantified, it is possible that many cats were FIV+ and FeLV+, as alluded to in theme 4 and 6, where responsible management has typically included the restriction of outdoor access to these cats.

### 4.8. Cat Autonomy

Most indoor-outdoor owners (94.4%) took into consideration that their cat indicated they wanted outdoor access when deciding on lifestyle. It was the major reason for providing this lifestyle for around a third of owners. Similarly, an Australian study found that 37% of owners who allowed their cat outdoor access at night did so for the cat’s freedom [53]. In comparison, 80.9% of indoor-only owners who considered an indoor-outdoor lifestyle considered if their cat indicated they wanted outdoor access. However, only 8.2% of indoor-only cats were ultimately reported to ‘choose’ their lifestyle by not leaving the house when able to. This leaves an overwhelming majority of 91.8% of indoor-only cats who may otherwise opt to roam outdoors if given the opportunity.

From this small percentage of cats opting not to go outside when given the choice, one may infer that most cats are highly motivated to access outdoor spaces if available. Some owners did report negative behavioural differences in their cats when restricting their outdoor access (themes 12 and 21), and indeed, undesirable behaviours are commonly reported as being more prevalent in indoor-only cats when compared to those with outdoor access [5,8,36,37,38]. Whilst enrichment items may provide the opportunity for cats to express natural behaviours indoors, the observed levels of undesirable behaviours in indoor-only cats might generally indicate the provision of suboptimal environments. A recent systematic review identified numerous gaps in the literature with regards to indoor-only cat welfare [73]. It also noted that some enrichment guidelines recommended by behaviourists or charities may not be evidence-based. This dearth of literature in the area may make it more difficult for owners to fully meet their cat’s behavioural needs within the home.

Currently, it is not known how the prevalence of undesirable behaviours, stress-related illnesses, or other welfare indicators vary between cats who choose to stay indoors, and those who have the choice made for them. It may be cats without the opportunity of choice are of an increased welfare concern. It has been posited that environmental control is beneficial for animals [74], and recent research into other domestic or captive species, including great apes, pandas, sheep, and goats, has demonstrated the positive impact of choice and control on welfare [75,76,77]. Further research into how choice and control may impact welfare in cats with owner-controlled and time-restricted outdoor access, as opposed to a freely accessible cat-flap, is therefore warranted.

### 4.9. Alternative Lifestyle

Most respondents did not consider an alternative lifestyle for their cat. This might suggest owners have an inherent view of appropriate cat husbandry they do not deter from. Such views are of potential concern if owners do not consider how individual temperaments or life experiences are suited to different lifestyles. However, it may transpire that owners did not consider an alternative lifestyle as they chose a cat deemed suitable for the lifestyle they wanted to provide. Further study into whether owners seek a suitable cat for their preferred lifestyle could indicate whether cats may be suffering due to inappropriate husbandry.

In this study, there was some indication owners may select a cat suitable for their chosen lifestyle. Some owners felt their cat’s personality was unsuitable for outdoor access (theme 9), indicating that they were making a judgement of their cats’ temperament and providing for them as they saw best. Other owners maintained the lifestyle their cat was used to, whether that be indoor-only or indoor-outdoor (themes 11 and 18). Additionally, owners indicated they were acting upon advice from veterinary professionals or rescue centres from which the cat was acquired (theme 8), although it is not evident whether this advice was based on temperament and lifestyle suitability or other factors such as safety or cat depredation.

When owners reported the major reason they would change their cat’s lifestyle, results echoed those of owners who had chosen the opposite lifestyle. For example, most indoor-only owners chose this lifestyle to protect their cat from traffic (58.7%), and most owners of indoor-outdoor cats reported if they were to change their cat’s lifestyle to indoor-only, it would be due to traffic (45.8%). Conversely, the benefits of outdoor access to mental health were acknowledged by many indoor-outdoor and indoor-only owners, with 38% and 35.3% giving this as the major reason for the lifestyle choice, or the reason they would change, respectively. This might suggest owners do recognise the positive and negative factors attributed to each lifestyle, even if they have a preferred lifestyle they adhere to.

Currently, we are unsure if there are differences between the management of indoor-only and indoor-outdoor cats with regards to resource provision, enrichment, and social interaction. If management varies, this could account for some of the perceived differences in the need for cats to obtain enrichment outdoors. For example, indoor-only cat owners who recognise the potential mental benefits of outdoor access may be more inclined to provide additional enrichment within the home when compared to those who do not acknowledge that outdoor access can be beneficial. Conversely, indoor-outdoor owners may feel that outdoor access is sufficiently enriching and provide less within the home, which could be problematic if they provide restricted outdoor access.

### 4.10. Limitations

As with any research, methodological limitations must be acknowledged. Online convenience sampling is a practical way of contacting large numbers of international cat owners; however, it may introduce sample bias. Owners chancing upon, and opting into, a survey regarding their cat may be systematically different from owners who do not find or engage in such surveys. This may be true for those who consent to participate in further studies and those who did or did not respond to the second survey. Generally, owners who did not participate may feel less strongly about the topic than owners who freely opted to give spare time for completing the surveys. This should be remembered when contemplating the strength of consideration owners assigned to different factors that influenced their decision making.

Additionally, it is acknowledged that this study does not present an exhaustive list of factors which may influence owner decision making. Other influences may include, but not limited to, previous cat ownership, place of cat acquisition, and age of cat at acquisition, as suggested by the thematic analysis. Because this study was the first detailed look at owner rationale for cat lifestyle, it is hoped that further studies can expand upon the results presented in this paper.

## 5. Conclusions

Ten owner and cat demographic variables were significantly associated with greater odds of cats being provided with either an indoor-only or indoor-outdoor lifestyle, inclusive of the global region, owner age, or cats having health issues, etc. Many of these variables offer evidence that urbanisation could be a driving factor behind the current data trends which suggest owners globally are moving towards indoor-only lifestyles for their cats. It was shown that owners living in city centres, urban environments, and flats/apartments were significantly more likely to have indoor-only cats. Strong concerns over traffic were voiced by indoor-only and indoor-outdoor cat owners, and RTAs were a major influencing factor for owners when deciding on lifestyle. Because urbanisation is set to continue, it is reasonable to assume that the proportion of indoor-only cats will continue to rise.

Considering the anticipated increase in indoor-only cats, alongside current literature suggesting indoor-only animals may exhibit more ‘undesirable’ and stress-linked sickness behaviours than indoor-outdoor cats, research focussing on how best to improve the behaviour and wellbeing of indoor-only cats would be beneficial. Particular attention should be paid to subgroups of cats found to be significantly more likely to be kept indoor-only, such as pedigree animals. Despite certain pedigree breeds being perceived as being better adapted to an indoor-only lifestyle, there is currently a paucity of scientific evidence in this area.

Finally, owners appeared to hold an inherent position in which they believe cats should have an indoor-only or indoor-outdoor lifestyle, as indicated by most owners not actively considering the alternative lifestyle. It is important for owners to recognise the individual needs of cats with different temperaments, activity requirements, or previous life experiences, lest the welfare of individuals suffer if not adequately provided for.

## Figures and Tables

**Table 1:** Owner demographics and their living environments of the 5129 respondents. Percentages for the entire group of respondents are shown, as are the breakdowns between those who indicated their cat had an indoor-only (*n* = 2104) or indoor-outdoor lifestyle (*n* = 3025).

Owner Demographics	Categories	Proportion of Total Population (%) (*n* = 5129)	Proportion of Indoor-Only Population (%) (*n* = 2104)	Proportion of Indoor-Outdoor Population (%) (*n* = 3025)
Owner gender	Female	89.1	87.9	89.9
	Male	9	9.5	8.7
	Other	1.2	1.9	0.7
	Prefer not to say	0.7	0.7	0.8
Owner age	18–25	14.1	14.3	14
	26–35	28.2	33.2	24.8
	36–45	23.7	23.2	24
	46–55	20	17.5	21.8
	56+	12.6	11.5	15
	Prefer not to say	0.4	0.3	0.5
Other cats	No	44.7	41.8	46.6
	Yes	55.3	58.2	53.4
Dogs	No	81.8	83.1	80.9
	Yes	18.2	16.9	19.1
Children (17 and under)	No	80.4	84.2	77.4
	Yes	19.6	15.2	22.6
Region	Europe	76.2	30.2	69.8
	USA and Canada	20.8	80.6	19.4
	AUS and NZ	3	42.2	57.8
Area	City centre	9.2	15.4	4.8
	Urban	20.1	24	17.4
	Suburban	41.9	38.9	44
	Village	16.9	11.5	20.7
	Rural	11.9	10.2	13.2
Dwelling Type	Flat/studio/apartment	20.6	37.5	8.8
	Terrace/town/row house	18.1	12.9	21.8
	Semi-detached	27.8	17.2	35.1
	Detached	27	26.2	27.5
	Bungalow/cottage	5.9	5.2	6.4
	Other	0.7	0.9	0.5

**Table 2:** Cat demographics and their management practices as reported by 5129 owners. The percentages for all cats can be seen, alongside a breakdown of those with an indoor-only lifestyle (*n* = 2104) and an indoor-outdoor lifestyle (*n* = 3025).

Cat Demographics	Categories	Proportion of Total Population (%) (*n* = 5129)	Proportion of Indoor-Only Population (%) (*n* = 2104)	Proportion of Indoor-Outdoor Population (%) (*n* = 3025)
Cat age	Kitten (0–6 months old)	1	1.7	0.4
	Junior (7 months–2 years old)	26	20.2	12.6
	Adult (3–6 years old)	33.1	43.3	43.3
	Mature (7–10 years old)	21.1	15.4	17.4
	Senior (11+ years old)	18.3	19	25.8
	Unsure	0.5	0.4	0.5
Cat sex	Female	50.6	51.9	49.6
	Male	49.3	48	50.2
	Unsure	0.1	0	0.2
Health problems	Yes	16.6	19.3	14.7
	No	83.4	80.7	85.3
Pedigree	Yes	11.2	16	7.9
	No	82.3	76.5	86.4
	Unsure	6.5	7.6	5.8
Neutered	Yes	96.8	95	98
	No	2.8	4.8	1.4
	Unsure	0.4	0.2	0.6
Microchipped	Yes	79	71.4	84.3
	No	19.8	27.4	14.5
	Unsure	1.2	1.2	1.2
Vaccinated	Yes	75.4	75.2	75.5
	No	20.8	21	20.7
	Unsure	3.9	3.9	3.9
Declawed	Yes	2.1	4.2	0.1
	No	97.9	95.7	99.4
	Unsure	0	0.1	0

**Table 3:** Model 1, owner demographics: Results of 4909 owner household variables tested through binary logistic regression for their association with cat lifestyle. Owners with increased odds of providing outdoor access are indicated by an odds ratio (OR) greater than one, whilst an OR lower than one indicates owners with increased odds of keeping cats as indoor-only.

Owner Household Variables	Sub-Group	Probability	OR	95% Confidence Interval (CI)
Owner gender	Female	Reference		
	Male	0.231	0.886	0.727–1.080
Owner Age	18–25	0.943	0.993	0.815–1.209
	26–35	0.001	0.765	0.651–0.898
	36–45	Reference		
	46–55	0.006	1.281	1.073–1.529
	56+	<0.001	1.499	1.224–1.836
Children	No	Reference		
	Yes	<0.001	1.707	1.461–1.995
Other cats	No	Reference		
	Yes	<0.001	0.768	0.683–0.865
Dog	No	Reference		
	Yes	0.078	1.149	0.984–1.340

**Table 4:** Model 2, cat demographics: Results of 4909 cat variables tested through binary logistic regression for their association with cat lifestyle. Cat features that increase their odds of being provided outdoor access are indicated by an OR greater than one, whilst an OR lower than one indicates cat features that increase odds of being kept as indoor-only.

Cat Variables	Sub-Group	Probability	OR	95% CI
Sex	Female	Reference		
	Male	0.016	1.155	1.028–1.298
Age	Junior	<0.001	0.656	0.565–0.762
	Adult	Reference		
	Mature	0.047	1.179	1.002–1.386
	Senior	<0.001	1.445	1.211–1.724
Pedigree	No	Reference		
	Unsure	0.004	0.707	0.559–0.894
	Yes	<0.001	0.441	0.367–0.529
Health Issues	No	Reference		
	Yes	<0.001	0.596	0.507–0.700

**Table 5:** Model 3, geographical features: Results of 4909 area variables tested through binary logistic regression for their association with cat lifestyle. Geographical variables where owners have increased odds of providing outdoor access are indicated by an OR greater than one, whilst an OR lower than one indicates geographical variables where owners have increased odds of keeping cats as indoor-only.

Area Variables	Sub-Group	Probability	OR	95% CI
Area	City centre	<0.001	0.442	0.341–0.574
	Urban	0.001	0.730	0.607–0.877
	Suburban	Reference		
	Village	0.796	0.974	0.801–1.186
	Rural	0.223	1.154	0.916–1.454
House Type	Flat/studio/apartment	<0.001	0.199	0.162–0.245
	Terrace/town/row house	0.165	0.868	0.711–1.060
	Semi-detached	Reference		
	Detached	0.385	1.093	0.894–1.336
	Bungalow/cottage	0.637	1.079	0.787–1.478
Region	Europe	Reference		
	USA and Canada	<0.001	0.093	0.076–0.114
	Australia and NZ	0.001	0.510	0.349–0.746

**Table 6:** The percentages of indoor-only cat owners (*n* = 1454) reporting different influence strength of factors on their decision to give their cats an indoor-only lifestyle and the major reasons for choosing an indoor-only lifestyle globally (*n* = 1333), then broken down by region.

Factors	Strength of Influence on Decision of Indoor-Only Cat Owners (*n* = 1454) (%)					Major Reason Lifestyle Was Chosen by Indoor-Only Owners (%)			
	None	Weak	Some	Moderate	Strong	Global (*n* = 1333)	USA & Can. (*n* = 634)	Europe (*n* = 645)	AUS & NZ (*n* = 54)
Prevent hunting	41.5	18	14.7	9.1	16.8	3.8	4.1	1.4	29.6
Protect from people	11.6	7.4	14.8	17.4	48.8	13.4	9.3	18.1	5.6
Protect from traffic	1.3	1.2	3.8	7	86.7	58.7	51.6	67.1	40.7
Protect from other cats	12.9	12.4	18.5	19.3	37	2.9	3.6	2.5	0
Protect from wildlife	19.9	13.4	13	13.9	39.8	10	19.9	0.5	7.4
Cat has health issues	79	6	4.6	3.1	7.3	2.6	1.9	3.3	1.9
Other	-	-	-	-	-	8.6	9.6	7.1	14.8

**Table 7:** Percentages of indoor-outdoor cat owners (*n* = 595) reporting different influence strength of factors considered during their decision to choose their cats’ lifestyle, with their major influencing reason. Percentages of consideration given by indoor-only owners who considered and an indoor-outdoor lifestyle for their cat (*n* = 131) and the percentage of indoor-only cat owners (*n* = 459) who gave different reasons when asked for the major factor that would cause them to change their cat to an indoor-outdoor lifestyle.

Factors	Strength of Influence on Decision by Indoor-Outdoor Cat Owners (*n* = 595) (%)					Major Reason Lifestyle Was Chosen (*n* = 594) (%)	Strength of Consideration by Indoor-only Cat Owners Who Considered an Indoor-Outdoor Lifestyle (*n* = 131) (%)					Major Reason Indoor-Only Owners Would Switch (*n* = 459) (%)
	None	Weak	Some	Moderate	Strong		None	Weak	Some	Moderate	Strong	
Mental health	1.7	1.2	19.5	6.9	70.8	38	3.1	3.1	16.8	19.8	57.3	35.3
Physical health	1.3	2.7	19.7	7.7	68.6	18	3.8	1.5	16.8	26.7	51.1	16.1
Toilets outside	23.9	12.6	14	17.3	32.3	5.4	42	16	14.5	10.7	16.8	5
Pest control	66.9	17.3	3.2	6.1	6.6	0.5	72.5	9.9	10.7	4.6	2.3	2.6
Cat wants outdoor access	5.6	3.7	13.5	8.6	68.7	32.9	19.1	13.7	22.9	18.3	26	24.4
Other	-	-	-	-	-	5.2	85.5	1.5	3.1	3.1	6.9	16.6

**Table 8:** Indoor-outdoor owners who considered an indoor-only lifestyle for their cat (*n* = 174) and the reported level of consideration given to different factors when making their decision and the percentage of indoor-outdoor cat owners (*n* = 593) who gave different reasons when asked for major factors that would cause them to change their cat to an indoor-only lifestyle would be.

Factors	Strength of Consideration by Indoor-Outdoor Cat Owners Who Considered an Indoor-Only Lifestyle (*n* = 174) (%)					Major Reason Indoor-Outdoor Owners Would Switch Lifestyle (*n* = 593) (%)
	None	Weak	Some	Moderate	Strong	
Prevent hunting	22.4	25.3	18.4	15.5	18.4	5.4
Protect from people	11.5	10.3	19.5	19	39.7	6.9
Protect from traffic	3.4	2.3	5.7	14.4	74.1	45.8
Protect from other cats	14.9	19.5	25.3	28.2	12.1	2.7
Protect from wildlife	32.2	28.7	19.5	10.3	9.2	0.5
Cat has health issues	75.3	8	6.3	2.3	8	34.9
Other	82.2	1.1	5.2	4.0	6.9	3.9

**Table 9:** Example quotes from owners used to create the 11 indoor-only rationale themes.

Theme	Example Comments
(1) Protection from traffic	‘Cats live near a busy road [...] afraid they get killed so keep indoors’; ‘Live on [a] main road and [my] previous cat got killed on [the] road’; ‘I would consider an indoor-outdoor lifestyle if we had a large garden […] and we lived away from busy roads’
(2) Protection from people	‘Previously had cat injured by [a] neighbour’; ‘I was advised dog fighting is prevalent in my area and cats are stolen as bait’; ‘She is a little blue-eyed cheetah and I worry she would get stolen’
(3) Protection from other animals	‘We have hawks that live in a large tree in our yard and have seen a coyote in our yard’; ‘She gets bullied by other cats’; ‘Feral cat colony outside and don’t want him exposed to disease’; ‘Next door neighbour’s Rottweiler killed a cat that went into their garden’
(4) Cat has health issues	‘Cat is deaf, so cannot safely go outside’; ‘Management of IBD’; ‘She has had a mammary carcinoma and requires regular medication each day’; ‘Cat is FIV+ and needs to be kept inside for his own safety and that of other cats’
(5) To protect wildlife	‘Domestic cats are a severe threat to birds’; ‘Impact of domestic and feral cats on bird and reptile populations’
(6) Protection from illness (*)	‘To prevent health issues often associated with outdoor animals, such as fleas, ticks, FIV, FIP, etc.’; ‘Fleas and ticks live outside. I do not want them in the house!’; ‘Outdoor exposure requires more aggressive flea/tick/other parasite treatment’; ‘Cat also eats outdoor toxic plants’
(7) To prevent getting lost (*)	‘Afraid she’d not find her way back’; ‘She was lost from her previous owners’ house (a few streets away!) for 3 years’; ‘Cat runs away to [their] previous house if let out (even after several years)’
(8) Acquisition requirement/recommendation (*)	‘Medical lab cat until 7.5 years old […] advised to keep indoor as would have no instinct for dangers’; ‘Signed agreement with breeder’; ‘Adoption agency contract specifies indoor-only’
(9) Personality unsuitable (*)	‘She’s also very skittish and I worry about her around traffic’; ‘He is very nervous and easily stressed’; ‘Our cat’s curious but too timid to stay outside for long’; ‘Too timid […] Shows no interest either’
(10) Pedigree cat (*)	‘Bengals seem notoriously “stupid” when it comes to keeping themselves safe if permitted free reign’; ‘Breed—Devon Rex—specifically bred as indoor cats’; ‘My cat is a breeding queen’
(11) Cat has no previous outdoor experience (*)	‘No outdoor experience when I got him. I don’t think he will have the necessary experience to keep safe’.

**Table 10:** Example quotes from owners used to create the 10 indoor-outdoor rationale themes.

Theme	Example Comments
(12) Beneficial to mental health	‘Would never have an indoor- only cat. Had one some years ago when I lived in a flat and he was a monster. Destroyed furniture, bedding, carpets, and clothing. When I moved to a house, he started to go outside and he calmed down completely’.
(13) Beneficial to physical health	‘Allows them to […] control their weight through increased exercise’; ‘My cat was kept in for 12 months but had IB symptoms i.e., diarrhoea. I think she was very stressed and unhappy as an indoor cat’.
(14) Cat indicates they want to go outside	‘I never force my cats either indoor or outdoor but let them make their own decision. This means they have their own choice which helps their mental wellbeing’; ‘I don’t want my cats to be captive—I want them to be free to choose to stay with us and to live as they choose’.
(15) Cat toilets outside	‘JLD will not toilet inside and becomes distressed if he has no outdoor access’; ‘I hate litter trays!’
(16) Pest control	‘I rely on their hunting to control rats and mice that would otherwise be attracted to the farmhouse and the chicken pens’.
(17) Enrichment (*)	‘Her world is so much bigger by having that access to the outdoors’; ‘Lots of interaction outside that I cannot provide indoors’; ‘My cat has always enjoyed sitting on the grass sniffing the fresh air’; ‘I hate the fact that one of my cats hunts […] However, they both find it distressing to be locked in’
(18) Previous outdoor access (*)	‘They were five when I got them and they had been used to going out’; ‘Most of my eight cats have been stray toms, a couple are still semi-feral, one of which gets very aggressive when kept in all the time’.
(19) Social opportunity (*)	‘I feel it’s unfair to leave my cat at home alone all day, when he could be outside and visiting the neighbours he really likes!’; ‘We enjoy having an active cat who engages with other cats in the neighbourhood’.
(20) Safe outdoor space (*)	‘I also do not live near a busy road so am happier letting them out.’; ‘We felt it was best for the cats, so bought appropriate properties’.
(21) Multi-cat household (*)	‘I live in a small house with four cats. A large garden gives the room to have a break from each other’; ‘It seemed unfair to have one rule for one cat and one rule for another’.
(22) Natural (*)	‘Allows natural behaviour for my cat’; ‘I feel that although humans, over the centuries, have domesticated cats, they still very much have a natural desire, interest in […] the outdoors’.

## Data Availability

The data presented in this study are available on request from the corresponding author. The data are not publicly available due to data privacy and identifying information of participants.
